# Impact of maternal hypothyroidism on human milk macronutrient content and fatty acid composition: a prospective cohort study

**DOI:** 10.3389/fnut.2026.1769344

**Published:** 2026-02-10

**Authors:** Shahad Alodhaybi, Manal Abdulaziz Binobaed, Rasha Homoud AlAnazi, Nora Elwehedy, Muneera Baraja, Fatimah Yousef Aljawoan, Waleed Tamimi, Lamia Mohammed Elamin, Azza Madkhali

**Affiliations:** 1 Department of Food Science and Nutrition, King Saud University, Riyadh, Saudi Arabia; 2 Department of Food Science and Nutrition, King Saud University, Riyadh, Saudi Arabia; 3 Pediatric Clinic, Ministry of National Guard Health Affairs, Riyadh, Saudi Arabia; 4 King Abdulaziz Medical City, King Abdullah International Medical Research Center, King Saud bin Abdulaziz University for Health Sciences, Riyadh, Saudi Arabia; 5 Family Medicine, Ministry of National Guard Health Affairs, Riyadh, Saudi Arabia; 6 Dent Medical Center, Riyadh, Saudi Arabia; 7 Breastfeeding Association, Riyadh, Saudi Arabia; 8 Laboratory Medicine, Ministry of National Guard Health Affairs, Riyadh, Saudi Arabia; 9 Consultant, Obstetrics and Gynecology, Ministry of National Guard Health Affairs, Riyadh, Saudi Arabia

**Keywords:** fatty acid composition, human milk, hypothyroidism, lactation, Saudi Arabia, trans fatty acids

## Abstract

**Background:**

Hypothyroidism can alter the serum lipid profile and the composition of human milk (HM) proteins involved in macronutrient metabolism. We investigated the association between maternal hypothyroidism and HM macronutrient content and fatty acid (FA) composition.

**Methods:**

In this prospective cohort study conducted in Riyadh, Saudi Arabia, HM samples from mothers with hypothyroidism (*n* = 19) and mothers without hypothyroidism (*n* = 30) were compared. Eligible participants were breastfeeding mothers of term singleton infants with no history of metabolic disorders or chronic disease. Maternal demographic characteristics, anthropometrics, laboratory markers, dietary intake, and HM samples were collected 4–13 weeks postpartum. Primary outcomes were HM macronutrient content and FA composition, analyzed using the Miris HM analyzer and gas chromatography–flame ionization detection, respectively.

**Results:**

Forty-nine participants were recruited between December 12, 2023, and May 31, 2025. HM macronutrient content and total saturated, monounsaturated, polyunsaturated, and trans FAs (TFAs) did not differ between groups. Among mothers with hypothyroidism, 10 individual FA species differed significantly (eight higher and two lower), characterized by higher industrial TFAs and selected long-chain omega-3 and omega-6 species. Mothers with hypothyroidism had higher levels of docosapentaenoic acid (0.079 ± 0.024% vs. 0.063 ± 0.024%) and elaidic acid (0.286% [0.233–0.439%] vs. 0.118% [0.028–0.220%]), but lower levels of tricosanoic acid (0.00% [0.00–0.00%] vs. 0.011% [0.00–0.022%]) (all *p* < 0.05). They also had higher low-density lipoprotein cholesterol (3.45 [2.87–3.97] vs. 2.94 [2.35–3.32] mmol/L; *p* = 0.01), whereas other lipid parameters did not differ significantly.

**Conclusion:**

Maternal hypothyroidism, even when treated with replacement therapy, was associated with altered HM FA composition. These changes may reflect thyroid hormone–related shifts in lipid metabolism, potentially influenced by maternal diet and adipose stores. Future longitudinal research is needed to investigate whether integrating thyroid-aware lactation support and targeted nutritional interventions can effectively modulate these effects and improve breastfeeding outcomes.

## 1 Introduction

The World Health Organization recommends initiating breastfeeding within the first hour of life, maintaining exclusive breastfeeding for 6 months, and continuing breastfeeding alongside complementary foods until 2 years of age or beyond (1). Given the unique biological properties of human milk (HM) and the dynamic, interactive nature of breastfeeding, breastfed infants and children are more likely to achieve optimal survival, growth, and development (2).

Maternal nutrition, weight, and health are key determinants of HM composition. In particular, maternal metabolic health, including obesity and diabetes, has been associated with differences in HM macronutrient levels and fatty acid (FA) composition (3–6). A systematic review and meta-analysis reported that maternal overweight/obesity is associated with higher HM fat content (3). A More recent study that sampled milk across a complete feeding (from foremilk to hindmilk) similarly found higher HM fat and energy content among women with overweight/obesity (4). Maternal obesity has also been linked to alterations in the HM FA profile, including lower proportions of docosahexaenoic acid (DHA) (5). Gestational diabetes mellitus (GDM) may also influence HM composition; a recent systematic review and meta-analysis reported differences in macronutrient content and highlighted emerging evidence of altered milk lipid profiles in mothers with GDM (6).

Thyroid hormones are critical regulators of maternal metabolism and contribute to cholesterol metabolism, *de novo* lipogenesis, FA oxidation, and carbohydrate metabolism. Insufficient thyroid hormone production, as observed in hypothyroidism, can disrupt these metabolic pathways (7). Although the global prevalence of hypothyroidism varies between 1 and 11%, studies in Saudi populations have reported higher rates, up to 44.5% (8–10). Maternal hypothyroidism has been associated with adverse pregnancy outcomes, including gestational complications, preterm birth, and impaired milk production, which may improve with thyroid replacement therapy (11, 12).

However, evidence on the effects of maternal hypothyroidism on HM composition remains limited. Animal studies suggest that untreated hypothyroidism during lactation impairs litter growth, reduces oxytocin levels, inhibits prolactin signaling, accelerates mammary involution, and alters milk composition, including reductions in triglycerides (TGs) and carbohydrates (13–15). In human studies, gestational hypothyroidism has been reported to alter colostrum and mature milk composition, including lower concentrations of proteins involved in macronutrient metabolism, increased immunoglobulin levels in colostrum, and reduced fat and protein content in mature milk (16, 17). Notably, these differences were reported despite thyroid replacement therapy.

Key limitations of the current literature include small sample sizes and limited characterization of the duration and/or severity of hypothyroidism exposure. FAs are among the most variable and functionally significant nutrient groups in HM and have essential roles in infant growth, immunity, and neurodevelopment (18). However, to the best of our knowledge, no studies have examined HM FA composition specifically in relation to maternal hypothyroidism. This gap is particularly relevant because thyroid hormones regulate multiple lipid metabolism pathways, suggesting a plausible link between maternal thyroid status and milk FA composition. Accordingly, the present study aimed to investigate the impact of maternal hypothyroidism on HM macronutrient and FA composition, with the goal of clarifying the role of maternal thyroid health in supporting infant nutrition.

## 2 Materials and methods

### 2.1 Study design and participants

This prospective cohort study included breastfeeding mothers residing in Riyadh, Saudi Arabia. Participants were recruited between December 2023 and May 2025 through two approaches: (1) in-person recruitment from pediatric clinics at primary care centers affiliated with King Abdulaziz Medical City and (2) online recruitment via breastfeeding support communities. A digital flyer was distributed via Child Care Association and Breastfeeding Association and included a link to an online eligibility questionnaire. Eligible mothers were contacted to schedule a visit to Dent Medical Center to complete the study.

**Figure 1:**
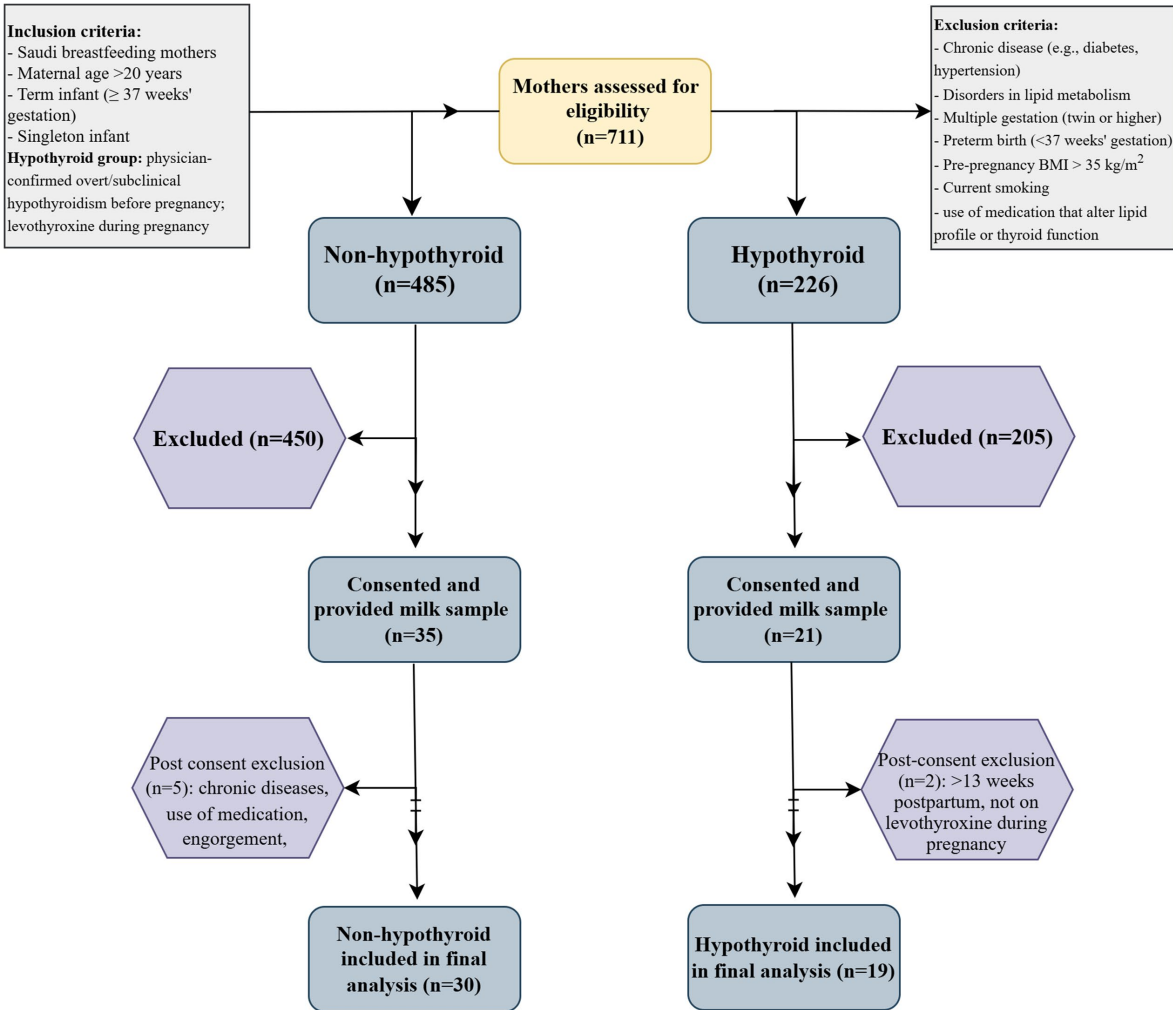
Recruitment flowchart for participants.

A total of 49 Saudi breastfeeding mothers participated and were assigned to either the hypothyroid group (*n* = 19) or the non-hypothyroid group (*n* = 30) (Figure 1). All assessments were completed during a single visit between 4 and 13 weeks postpartum.

Eligible participants were Saudi breastfeeding mothers aged ≥20 years with term singleton infants. Mothers were excluded if they had metabolic disorders related to lipid metabolism; chronic diseases such as hypertension or diabetes; morbid obesity prior to pregnancy (body mass index [BMI] > 35 kg/m^2^); smoking; or use of medications known to affect the lipid profile or thyroid function.

### 2.2 Hypothyroidism and levothyroxine treatment

Mothers were classified as hypothyroid if they had a physician-confirmed diagnosis of overt or subclinical hypothyroidism before pregnancy and were prescribed levothyroxine throughout pregnancy and the postpartum period (12). Diagnosis status and levothyroxine dosage were verified through medical records. Information on thyroid hormone therapy, including dosage and adherence, was collected from mothers with hypothyroidism using a structured maternal questionnaire.

Mothers were asked about levothyroxine use during the preceding 4 weeks, dosing frequency (daily, <3 times/week, 4–5 times/week, or never), and whether levothyroxine was taken before laboratory testing. All hypothyroid mothers used oral levothyroxine tablets, with formulation determined by prescribed dose: Euthyrox® (levothyroxine sodium; Merck KGaA, Darmstadt, Germany) for the 25-μg dose and Eltroxin® (levothyroxine sodium; Aspen Pharma Trading Ltd., Dublin, Ireland) for the 50-μg and 100-μg doses. Thyroid status at the time of milk collection was assessed using serum thyroid-stimulating hormone (TSH) concentrations and categorized as below, within, or above the reference range.

### 2.3 Maternal and infant anthropometric measurements

Maternal height in centimeters (cm) was measured using a Seca stadiometer (Seca, Hamburg, Germany). Body weight and body composition (fat percentage, muscle mass, and BMI) were assessed using a TANITA BC-545F bioimpedance scale (Tanita Corporation, Tokyo, Japan). Pre-pregnancy BMI was calculated using recalled pre-pregnancy weight and verified against medical records. BMI was calculated as weight in kilograms (kg) divided by height in meters squared (m^2^) (kg/m^2^). Infant weight, length, and head circumference were measured by a pediatric nurse during the routine 2-month well-baby visit. Birth weight was obtained from medical records.

### 2.4 Maternal demographics and dietary information

A structured questionnaire was used to collect maternal demographic characteristics (age, employment status, educational level, and household income) and medical history (pre-pregnancy weight, parity, mode of delivery, breastfeeding history, and use of medications and supplements). Current breastfeeding practices were assessed using a questionnaire, and infants were classified as exclusively breastfed or receiving mixed feeding; for mixed feeding, the number of formula feeds per day was recorded. Dietary intake was assessed using interviewer-administered 24-h dietary recalls collected on 3 days (2 weekdays and 1 weekend day) to evaluate macronutrient and energy intake. Dietary data were analyzed using ESHA Food Processor software (ESHA Research, Salem, OR, USA). Traditional Saudi foods were entered as individual ingredients to support nutrient calculations.

### 2.5 Sample collection and analysis

#### 2.5.1 Blood samples

Maternal blood samples were collected during the visit to evaluate thyroid function (TSH, free triiodothyronine [FT3], free thyroxine [FT4], and thyroid peroxidase antibodies [TPOAb]) and lipid profile parameters (total cholesterol [TC], low-density lipoprotein cholesterol [LDL-C], high-density lipoprotein cholesterol [HDL-C], and TGs). Venous blood samples were collected without a fasting requirement, and fasting status and levothyroxine intake were documented at the time of collection. Thyroid function was analyzed using a chemiluminescent immunoassay on an Alinity i system, and lipid profile measurements were performed on an Alinity c clinical chemistry analyzer using enzymatic colorimetric methods (Abbott Laboratories, IL, USA).

#### 2.5.2 Milk sample

HM samples were collected between 4 and 13 weeks postpartum, a period selected based on the expected increase in milk supply and transition into mature milk. Samples were obtained by full expression of one breast at least 2 h after the last feeding to capture both foremilk and hindmilk. Collection was performed between 09:00 and 15:00 using a hospital-grade pump (Symphony®, Medela) to minimize variability. After gentle mixing, a 20–30 mL aliquot was collected for analysis, and the remaining milk was returned to the mother. Samples were temporarily refrigerated and transported in insulated cooler bags to Ajal laboratories for storage at −70 °C.

### 2.6 Fatty acid analysis

FA analysis was conducted within 3 months of sample collection. Prior to analysis, samples were thawed at room temperature. Fat was extracted according to the gravimetric ISO 1736:2008(E) IDF 9:2008(E) method (19), with minor modifications for liquid samples validated through interlaboratory comparisons. Extractions were performed in triplicate to support analytical precision.

A 0.1-g aliquot of extracted fat was converted into FA methyl esters (FAMEs) using sequential base- and acid-catalyzed reactions (methanolic KOH followed by sulfuric acid in methanol). The resulting FAMEs were extracted in n-heptane and analyzed using gas chromatography with flame ionization detection on an SP-2560 capillary column (100 m × 0.25 mm ID, 0.2-μm film thickness; Restek), with helium as the carrier gas. Injector and detector temperatures were set at 250 °C, with a split ratio of 1:50. The oven temperature was increased at 3.0 °C per minute to 240 °C, followed by a final hold of 24 min. FAMEs were identified by comparing retention times with external standards (Supelco 37 Component FAME Mix plus 13 trans-FAME mix; Supelco, Merck/Sigma-Aldrich, St Louis, MO, USA). FAs were expressed as a percentage of total identified FAs (% area).

### 2.7 Macronutrient analysis

HM macronutrients (fat, protein, and carbohydrate) and total solids were measured using a Miris HM Analyzer (Miris AB, Uppsala, Sweden). Energy content was determined by the analyzer based on macronutrient composition. The instrument uses mid-infrared transmission spectroscopy and is calibrated against standardized reference methods. Prior to analysis, HM samples were thawed at room temperature, homogenized using a Miris Ultrasonic Processor, and heated in a 40 °C water bath.

### 2.8 Statistical analysis

Data were analyzed using the Statistical Package for Social Sciences (SPSS) software (version 27.0; IBM Corp., Armonk, NY, USA). Before formal analysis, data were verified for completeness and accuracy. Categorical variables were compared using Pearson’s chi-square test, and Fisher’s exact test was used when expected cell counts were <5. Continuous variables are summarized as mean ± standard deviation, and categorical variables are expressed as frequencies and percentages.

Normality of continuous variables was assessed using the Shapiro–Wilk test. Group comparisons were performed using the independent-samples *t*-test for normally distributed variables and the Mann–Whitney *U* test for non-normally distributed variables. A *p*-value < 0.05 was considered statistically significant. To assess the potential influence of breastfeeding practices, analysis of covariance (ANCOVA) was performed for FAs that showed statistically significant differences, with breastfeeding exclusivity entered as a covariate. Correlations between thyroid function and milk FA composition were assessed using Pearson’s correlation.

## 3 Results

### 3.1 Demographic characteristics

Table 1 presents the demographic characteristics of the study participants. Maternal age, education level, employment status, household income, history of gestational diabetes, mode of delivery, omega-3 (n-3) and multivitamin supplement use, parity, and breastfeeding history did not differ between the hypothyroid and non-hypothyroid groups (*p* > 0.05 for all). More than half of the participants reported multivitamin use in the previous 4 weeks. Reported n-3 intake during pregnancy was low (16%), and only one mother reported postpartum n-3 use. Infant sex and intake of herbal drinks also did not differ between groups.

**Table 1:** Descriptive characteristics of hypothyroid and non-hypothyroid mothers.

Characteristic	Non-hypothyroid (*n* = 30)	Hypothyroid (*n* = 19)	*p* value
Maternal age (years)			0.41
20–25	4 (13.3%)	0 (0.0%)	
26–30	5 (16.7%)	6 (31.6%)	
31–35	9 (30.0%)	5 (26.3%)	
36–40	7 (23.3%)	6 (31.6%)	
>40	5 (16.7%)	2 (10.5%)	
Education			0.12
Not educated	2 (6.7%)	0 (0.0%)	
Highschool or less	9 (30.0%)	7 (36.8%)	
Diploma	0 (0.0%)	3 (15.8%)	
University education	18 (60.0%)	9 (47.4%)	
Higher education	1 (3.3%)	0 (0.0%)	
Employment status			0.41
Employed	3 (10.0%)	4 (21.1%)	
Not employed	27 (90.0%)	15 (78.9%)	
Household income			0.76
<5,000	2 (6.7%)	0 (0.0%)	
5,001–10,000	15 (50.0%)	10 (52.6%)	
10,001–15,000	9 (30.0%)	5 (26.3%)	
>15,001	4 (13.3%)	4 (21.1%)	
Omega-3 during pregnancy			0.15
Yes	6 (20.0%)	2 (10.5%)	
No	24 (80.0%)	17 (89.5%)	
Omega-3 supplements (last 4 weeks)			1.00
Yes	1 (3.3%)	0 (0.0%)	
No	29 (96.7%)	19 (100.0%)	
Multivitamin supplement			0.22
Yes	18 (60.0%)	8 (42.1%)	
No	12 (40.0%)	11 (57.9%)	
Multivitamin intake frequency			0.29
Less than 3 times/week	5 (16.7%)	0 (0.0%)	
4–5 times/week	4 (13.3%)	2 (10.5%)	
Daily	9 (30.0%)	6 (31.6%)	
Parity			0.56
1 child	7 (23.3%)	2 (10.5%)	
2 children	5 (16.7%)	6 (31.6%)	
3 children	3 (10.0%)	2 (10.5%)	
4+ children	15 (50.0%)	9 (47.4%)	
GDM			0.69
Yes	5 (16.7%)	2 (10.5%)	
No	25 (83.3%)	17 (89.5%)	
Mode of delivery			0.71
Vaginal	22 (73.3%)	13 (68.4%)	
Caesarean section	8 (26.7%)	6 (31.6%)	
History of breastfeeding			0.32
Yes	21 (70.0%)	16 (84.2%)	
No	9 (30.0%)	3 (15.8%)	
Duration of previous breastfeeding			0.06
<6 months	4 (19.0%)	9 (56.3%)	
6–11 months	2 (9.5%)	2 (12.5%)	
1–1.5 years	7 (33.3%)	1 (6.3%)	
2 years or less	8 (38.1%)	4 (25.0%)	
Infant sex			0.88
Boy	12 (40.0%)	8 (42.1%)	
Girl	18 (60.0%)	11 (57.9%)	
Infant herb intake			0.21
Yes	10 (33.3%)	3 (15.8%)	
No	20 (66.7%)	16 (84.2%)	

Data are *n* (%). Values are compared between groups using *χ*^2^ test or Fisher’s exact test, as appropriate. GDM, gestational diabetes mellitus.

Maternal anthropometric characteristics, including pre-pregnancy BMI, postpartum BMI, fat mass percentage, muscle mass (kg), and body water percentage, were comparable between groups. Infant measurements (birth weight, 2-month weight, length, and head circumference) were also comparable (Supplementary Table S1).

### 3.2 Maternal diet

Average dietary intake of energy, macronutrients, and most FAs did not differ between groups (Supplementary Table S2). Mothers with hypothyroidism reported higher polyunsaturated FAs (PUFAs) intake than mothers without hypothyroidism (16.8 ± 5.2 vs. 13.5 ± 5.8 g/day; *p* = 0.050). Intakes of n-3, omega-6 (n-6), and the n-6/n-3 ratio did not differ between groups (*p* > 0.05 for all).

### 3.3 Thyroid status and lipid profile

At the time of milk collection, more than half of mothers with hypothyroidism had TSH concentrations outside the reference range. Most mothers with hypothyroidism (94.7%) reported levothyroxine use in the preceding 4 weeks, although three reported inconsistent adherence (Supplementary Table S3). TPOAb positivity was detected in 94.7% of mothers with hypothyroidism and 36.7% of mothers without hypothyroidism (Supplementary Table S4). The only mother with hypothyroidism who tested negative for TPOAb had undergone total thyroidectomy.

Plasma LDL-C concentrations were higher among mothers with hypothyroidism than among those without hypothyroidism (3.45 [2.87–3.97] vs. 2.94 [2.35–3.32] mmol/L; *p* = 0.01). HDL-C, TC, and TG levels did not differ between groups (Table 2).

**Table 2:** Comparison of maternal lipid profile between hypothyroid and non-hypothyroid mothers.

Lipid profile	Non-hypothyroid (*n* = 30)	Hypothyroid (*n* = 19)	*P* value
Total cholesterol (mmol/L)	4.79 ± 0.68	5.12 ± 0.79	0·06
TG (mmol/L)	1.17 ± 0.81	0.91 ± 0.39	0·75
LDL (mmol/L)	2.94 ± 0.68	3.53 ± 0.97	**0.01**
HDL (mmol/L)	1.46 ± 0.32	1.41 ± 0.27	0.63

Data are mean (SD). *p* values for continuous variables are from two-sample *t* test or Mann–Whitney *U* test, depending on distribution. TG, triglycerides. LDL, low-density lipoprotein cholesterol. HDL, high-density lipoprotein cholesterol. Bold values indicate statistical significance (*p* < 0.05).

### 3.4 Human milk macronutrients and fatty acids

Tables 3 and 4 summarize HM macronutrient content and FA composition, respectively, in mothers with and without hypothyroidism. A total of 47 FAs were identified, including SFAs, MUFAs, PUFAs, and trans FAs (TFAs). Oleic acid (C18:1 n-9) and palmitic acid (C16:0) were the most abundant FAs.

**Table 3:** Comparison of milk macronutrients between hypothyroid and non-hypothyroid mothers.

Milk	Non-hypothyroid (*n* = 30)	Hypothyroid (*n* = 19)	*P* value
Carbohydrates (g/100 mL)	7.6 ± 0.6	7.8 ± 0.3	0.92
Fat (g/100 mL)	4.1 ± 1.7	3.4 ± 1.1	0.18
True protein (g/100 mL)	1.1 ± 0.5	1.1 ± 0.3	0.81
Crude protein (g/100 mL)	1.4 ± 0.6	1.3 ± 0.4	0.83
Total solids (g/100 mL)	13.2 ± 1.8	12.7 ± 1.2	0.42
Energy (kcal/100 mL)	74.2 ± 16.1	68.1 ± 10.4	0.33

Data are mean (SD). *P* values for continuous variables are from Mann–Whitney *U* test.

**Table 4:** Comparison of milk fatty acids between hypothyroid and non-hypothyroid mothers.

Fatty acids %	Non-hypothyroid (*n* = 30)	Hypothyroid (*n* = 19)	*P* value
Total SFAs	46.238 ± 6.474	47.157 ± 7.113	0.643
C6:0 (Hexanoate)	0.021 ± 0.016	0.029 ± 0.014	0.078
C8:0 (Octanoate)	0.117 ± 0.058	0.144 ± 0.060	0.125
C10:0 (Decanoate “caprate”)	1.290 ± 0.394	1.299 ± 0.454	0.938
C11:0 (Undecanoate)	0.002 ± 0.004	0.007 ± 0.008	**0.002**
C12:0 (Laurate)	6.602 ± 2.824	6.124 ± 2.883	0.637
C13:0 (Tridecanoate)	0.015 ± 0.015	0.019 ± 0.010	0.187
C14:0 (Myristate)	7.086 ± 3.446	6.612 ± 2.807	0.870
C15:0 (Pentadecanoate)	0.203 ± 0.081	0.237 ± 0.080	0.152
C16:0 (Palmitate)	25.767 ± 2.608	25.044 ± 1.859	0.552
C17:0 (Heptadecanoate)	0.227 ± 0.076	0.256 ± 0.072	0.189
C18:0 (Stearate)	5.286 ± 0.766	5.429 ± 0.650	0.821
C20:0 (Arachidate)	0.143 ± 0.044	0.140 ± 0.026	0.810
C21:0 (Heneicosanoate)	0.111 ± 0.058	0.158 ± 0.073	**0.017**
C22:0 (Behenate)	0.062 ± 0.032	0.049 ± 0.013	0.185
C23:0 (Tricosanoate)	0.012 ± 0.012	0.002 ± 0.006	**0.002**
C24:0 (Lignocerate)	0.033 ± 0.021	0.033 ± 0.011	0.850
Total MUFAs	37.043 ± 4.992	35.221 ± 4.627	0.207
C14:1 (Myristoleic acid)	0.120 ± 0.069	0.158 ± 0.070	0.068
C16:1 (Palmitoleate)	2.358 ± 1.066	2.465 ± 0.866	0.715
C17:1 (Heptadecenoic acid)	0.111 ± 0.085	0.104 ± 0.090	0.827
C18:1 n-9 (Oleic acid)	32.399 ± 4.343	31.773 ± 3.633	0.603
C18:1 n-7 (Vaccenic acid)	0.736 ± 0.328	0.891 ± 0.290	0.098
C20:1 n-9 (Gondoic)	0.289 ± 0.082	0.311 ± 0.041	0.076
C22:1 n-9 (Erucate)	0.044 ± 0.019	0.047 ± 0.009	0.199
C24:1 n-9 (Nervonate)	0.026 ± 0.011	0.028 ± 0.009	0.659
Total PUFA	15.911 ± 2.756	16.481 ± 3.812	0.547
C18:2 n-6 (Linoleic acid)	13.939 ± 2.813	14.802 ± 3.665	0.566
C18:3 n-6 (γ-Linolenic acid, GLA)	0.122 ± 0.050	0.153 ± 0.065	0.068
C18:3 n-3 (α-Linolenic acid, ALA)	0.369 ± 0.152	0.431 ± 0.144	0.079
C18:4 n-3 (Stearidonic acid, SDA)	0.011 ± 0.019	0.004 ± 0.009	0.753
C20:2 (Eicosadienoic acid)	0.258 ± 0.063	0.266 ± 0.050	0.218
C20:3 n-6 (Dihomo-γ-linolenic acid, DGLA)	0.459 ± 0.149	0.484 ± 0.105	0.207
C20:3 n-3 (11,14,17-Eicosatrienoic acid)	0.004 ± 0.007	0.012 ± 0.009	**0.002**
C20:4 n-6 (Arachidonic acid, ARA)	0.459 ± 0.101	0.478 ± 0.106	0.537
C22:2 n-6 (Docosadienoic acid)	0.064 ± 0.018	0.079 ± 0.023	**0.011**
C20:5 n-3 (Eicosapentaenoic acid, EPA)	0.016 ± 0.010	0.021 ± 0.010	0.110
C22:5 n-3 (Docosapentaenoic acid, DPA)	0.063 ± 0.024	0.079 ± 0.024	**0.023**
C22:6 n-3 (Docosahexaenoic acid, DHA)	0.082 ± 0.042	0.099 ± 0.041	0.083
Total TFA	0.273 ± 0.169	0.399 ± 0.253	0.065
C14:1 (Myristoleic acid, trans)	0.004 ± 0.014	0.004 ± 0.014	0.984
C15:1 (Pentadecenoic acid, trans)	0.006 ± 0.017	0.006 ± 0.017	0.840
C16:1 (Palmitoleate, trans)	0.063 ± 0.049	0.102 ± 0.047	**0.011**
C17:1 (Heptadecenoic acid, trans)	0.001 ± 0.004	0.003 ± 0.007	0.505
C18:1 n-9 (Elaidic acid)	0.145 ± 0.129	0.315 ± 0.172	**0.001**
C18:1 (Vaccenic acid, trans)	0.021 ± 0.047	0.005 ± 0.020	0.064
C18:2 n-6 (Linolelaidic acid)	0.005 ± 0.010	0.003 ± 0.008	0.782
C19:1 (7-Nonadecenoate, trans)	0.060 ± 0.066	0.129 ± 0.060	**0.001**
C19:1 (10-Nonadecenoate, trans)	0.027 ± 0.043	0.007 ± 0.021	**0.040**
C20:1 (Gondoic acid, trans)	0.002 ± 0.004	0.001 ± 0.004	0.879
Omega-3	0.505 ± 0.174	0.559 ± 0.221	0.349
Omega-6	15.150 ± 2.748	15.675 ± 3.678	0.571
Omega-7	0.625 ± 0.361	0.732 ± 0.308	0.289
Omega-9	33.946 ± 4.668	31.647 ± 4.118	0.086
Omega-6/Omega-3	34.620 ± 16.649	31.857 ± 13.886	0.782

Data are mean (SD). *p* values for continuous variables are from two-sample *t* test or Mann–Whitney *U* test, depending on distribution. SFA, saturated fatty acids; MUFA, monounsaturated fatty acids; PUFA, polyunsaturated fatty acids; TFA, trans fatty acids. Bold values indicate statistical significance (*p* < 0.05).

Total HM macronutrients and overall FA classes did not differ between groups; however, several individual FAs differed. Tricosanoic acid (C23:0) and 10-nonadecenoate (C19:1 trans-9) were higher in milk from mothers without hypothyroidism. By contrast, milk from mothers with hypothyroidism had higher undecanoic acid (C11:0), heneicosanoic acid (C21:0), cis-11,14,17-eicosatrienoic acid (C20:3 n-3), cis-13,16-docosadienoic acid (C22:2 n-6), docosapentaenoic acid (C22:5 n-3; DPA), palmitelaidic acid (C16:1 trans-9), elaidic acid (C18:1 n-9 trans), and nonadecenoic acid (C19:1 trans-7) (*p* < 0.05). To verify that the observed differences were not driven by feeding practices, we performed an ANCOVA adjusting for breastfeeding exclusivity. The analysis confirmed that breastfeeding exclusivity was not a significant covariate (*p* > 0.05 for all comparisons) and did not alter the significant association between maternal thyroid status and FA profiles (Supplementary Table S5).

### 3.5 Correlation between thyroid function and fatty acids

**Figure 2:**
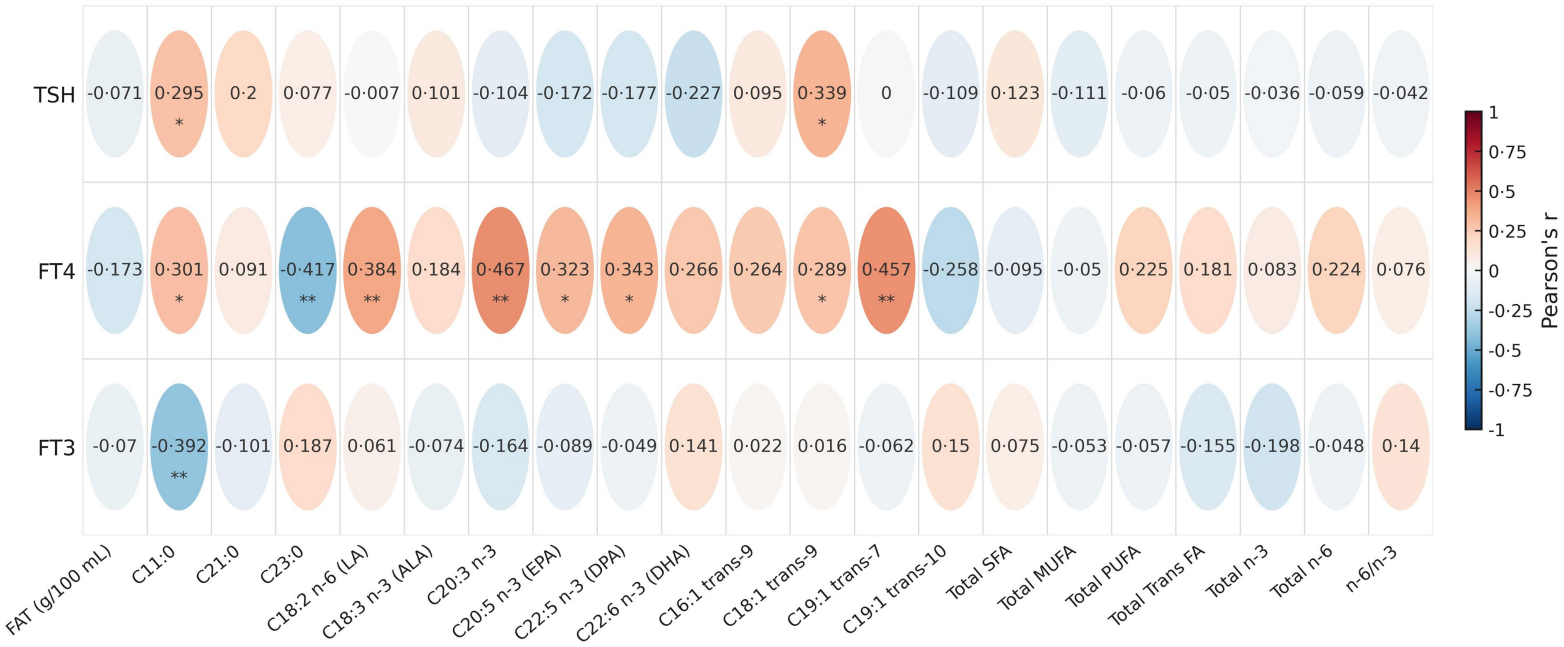
Pearson’s correlation between thyroid hormones (TSH, FT4, and FT3) and human milk fatty acids (*N* = 49). Two-tailed tests * and ** indicate *p* < 0.05 and *p* < 0.01, respectively. Colors indicate correlation strength and direction (blue: negative to red: positive). FAT, fat concentration (g/100 mL); SFA, saturated fatty acids; MUFA, monounsaturated fatty acids; PUFA, polyunsaturated fatty acids; ALA, *α*-linolenic acid; EPA, eicosapentaenoic acid; DPA, docosapentaenoic acid; DHA, docosahexaenoic acid; TSH, thyroid-stimulating hormone; FT3, free triiodothyronine; FT4, free thyroxin.

Correlations between thyroid function measures and HM FA levels were assessed for total FAs, biologically important FAs, and FAs that differed between groups (Figure 2; Supplementary Table S6). FT4 was positively correlated with C11:0, linoleic acid (C18:2 n-6; LA), C20:3 n-3, C22:2 n-6, eicosapentaenoic acid (C20:5 n-3; EPA), DPA, C18:1 trans-9, and C19:1 trans-7, and negatively correlated with C23:0. TSH was positively correlated with C11:0 and C18:1 trans-9, whereas FT3 was negatively correlated with C11:0. Complete correlation results for all identified FAs are provided in Supplementary Table S6.

## 4 Discussion

In this study, mothers with hypothyroidism showed differences in the FA composition of HM despite broadly comparable demographic characteristics, anthropometric measures, and reported macronutrient and energy intake. Although mothers with hypothyroidism reported higher total PUFA intake, reported n-3 and n-6 intakes did not differ between groups. HM macronutrients (fat, protein, and carbohydrates) and the overall distribution of SFA, MUFAs, PUFAs, and TFAs were also comparable. However, several individual FAs differed between groups (Tables 3 and 4). Specifically, among SFAs, C11:0 and C21:0 were higher, and C23:0 was lower in the hypothyroid group. Within the PUFA class, mothers with hypothyroidism exhibited higher levels of C20:3 n-3, C22:2 n-6, and DPA. Additionally, they had higher concentrations of several TFAs, including C16:1 trans-9, C18:1 n-9 trans, and C19:1 trans-7, with a concurrent decrease in C19:1 trans-9 levels. These differences co-occurred with higher plasma LDL-C concentrations in mothers with hypothyroidism.

Correlation analyses further supported associations between thyroid function markers and several milk FAs, with free thyroxine (FT4) showing the most consistent relationships (Figure 2; Supplementary Table S6). FT4 was positively associated with several FAs that differed between groups (including C11:0, C20:3 n-3, C22:2 n-6, DPA, C18:1 n-9 trans, and C19:1 trans-7) and negatively associated with C23:0; FT4 was also positively associated with linoleic acid (C18:2 n-6; LA) and eicosapentaenoic acid (C20:5 n-3; EPA).

Although the proportions of SFAs, MUFAs, and C18:1 n-9 in our cohort were consistent with global HM FA profiles, total PUFA, total n-3, and DHA were lower than global averages (20). This pattern was reflected in the HM n-6/n-3 ratio (% of total FAs), which was high in both groups (34.62 ± 16.65 in the non-hypothyroid group and 31.86 ± 13.89 in the hypothyroid group). For context, a systematic review and meta-analysis reported pooled mean concentrations of n-6 and n-3 FAs in mature milk of 13.59 and 1.02%, respectively, corresponding to an implied n-6/n-3 ratio of approximately 13.3 (21). In our cohort, the elevated HM ratio was consistent with the 3-day dietary recalls, which indicated low consumption of n-3–rich foods and frequent use of n-6–rich vegetable oils. Only two women consumed oily fish (salmon), three consumed other fish, and six consumed canned tuna during the recall period, whereas corn and sunflower oils were commonly used. This pattern is characteristic of the study’s inland location in Saudi Arabia, where seafood consumption is traditionally lower compared to the coastal regiones. Consistent with these patterns, the dietary n-6/n-3 ratio was 12.07 ± 5.76 in the non-hypothyroid group and 13.89 ± 5.91 in the hypothyroid group. Importantly, HM FA composition reflects not only current intake but also longer-term dietary patterns and mobilization of endogenous lipid stores (22–24). This is illustrated by a 10-day crossover supplementation study comparing CLA-rich Alpine butter with margarine: despite a large difference in the n-6/n-3 ratio of the intervention fats (≈1 in butter vs. ≈ 50 in margarine), the milk n-6/n-3 ratio changed only modestly (13.7 vs. 17.9), highlighting that milk FA composition is not exclusively driven by short-term intake (25).

In the present study, milk macronutrient content did not differ between groups. This contrasts with Jin et al., who reported lower fat and protein levels in milk from mothers with gestational hypothyroidism (17). Differences in study populations, exposure characterization, sampling approaches, and sample size may contribute to the discrepancy. In a related proteomic analysis, milk from mothers with gestational hypothyroidism showed no difference in the total number of proteins identified; however, multiple proteins involved in macronutrient metabolism were downregulated (16). To our knowledge, these are the only human studies evaluating gestational hypothyroidism and HM macronutrient composition. Although human data on hypothyroidism are scarce, a large body of evidence exists on other metabolic conditions, such as overweight/ obesity and GD. Results vary across individual studies; however, recent systematic reviews and meta-analyses indicate that milk from mothers with obesity often exhibits higher fat content, whereas milk from mothers with GD tends to have higher protein and fat levels (3, 6).

In animal models, reduced levels of thyroid hormones have been associated with lower TG content in milk and impaired growth in offspring, underscoring the physiological importance of hypothyroidism on lactational outcomes and development (14). HM fat is secreted as milk fat globules containing a TG-rich core enveloped by a membrane. These globules are synthesized within mammary epithelial cells prior to secretion into milk (26). HM FAs originate from *de novo* synthesis in the mammary gland, maternal diet, and maternal adipose tissue (20). The pattern of altered SFAs, PUFAs, and TFAs observed here aligns with reports linking thyroid dysfunction to shifts in circulating lipid species and serum FA profiles (27, 28). Jin et al. also reported smaller milk fat globule size in mothers with hypothyroidism, raising the possibility that altered mammary lipid handling may contribute to differences in milk fat composition (17). Notably, the FA differences observed in our study occurred among mothers treated with thyroid hormone replacement therapy, and a substantial proportion of mothers with hypothyroidism had TSH concentrations outside the reference range at sampling.

Similar complexity has been described in other maternal metabolic disorders. Studies comparing HM FA composition by maternal BMI have reported differences in SFAs, MUFAs, and PUFAs (29–31). Higher maternal BMI has been associated with higher SFAs and a higher n-6/n-3 ratio, whereas n-3 PUFAs (including DHA) and the precursor *α*-linolenic acid (ALA) are often lower in milk from mothers with obesity (29, 31–34). HM from mothers with GDM has also been reported to show altered concentrations of specific n-6 FAs even when total SFAs, MUFAs, and PUFAs are comparable with controls (35). Collectively, these findings suggest that maternal metabolic dysregulation may be associated with selective shifts in HM FA profiles rather than uniform changes across lipid classes.

Although maternal diet can influence HM FA composition, evidence from other metabolic states suggests that differences may persist even with similar reported intake. For example, studies on maternal obesity found that even at comparable levels of n-3 (DHA) intake, DHA levels remained lower in the milk of women with obesity (5, 36). Together, these observations support the interpretation that the FA differences observed in this study may reflect differences in maternal metabolic state in addition to reported dietary intake.

Several mechanisms may contribute, including thyroid hormone regulation of enzymes involved in lipid metabolism and hypothyroidism-associated inflammation. Thyroid hormones modulate genes involved in *de novo* lipogenesis (e.g., acetyl-CoA carboxylase [ACC] and fatty acid synthase) and influence hepatic lipase and lipoprotein lipase (LPL), thereby affecting lipolysis (7, 37). In animal studies, Hapon et al. reported reduced ACC activity in liver and mammary tissue, lower mammary ACC and LPL mRNA levels during late lactation, and reduced hepatic ACC mRNA levels (14). Hypothyroidism has also been associated with systemic inflammation. This inflammatory pathway appears to be a shared mechanism across maternal metabolic disorders; consistent with the chronic low-grade inflammation seen in obesity and GD, hypothyroidism is associated with noted elevated cytokine levels and inflammatory markers (28, 38). Chronic inflammation may adversely affect adipose LPL activity, potentially restricting the availability of some PUFAs or long-chain FAs (39).

Milk TFAs reflect intake and mobilized endogenous stores from ruminant fats and industrial partially hydrogenated oils (PHOs). TFAs are generally considered unfavorable because they can interfere with enzymes involved in long-chain PUFA synthesis, potentially affecting essential FA availability in HM and infant circulation (40). However, TFAs differ by isomer/source: ruminant-derived TFAs, such as vaccenic acid, have been suggested to have different biological effects than industrial TFAs, such as elaidic acid, which are consistently associated with adverse health outcomes (40). In this context, higher levels of TFAs such as elaidic acid in the hypothyroid group may reflect differences in dietary sources and/or differential mobilization of stored TFAs, consistent with an interaction between thyroid status and lipid metabolism; however, the present study design does not allow source attribution.

Consistent with the lipid metabolism role of thyroid hormones, LDL-C concentrations were higher in mothers with hypothyroidism, without corresponding differences in TG, HDL-C, or total cholesterol. Prior studies have similarly reported elevated LDL-C in hypothyroidism (41, 42), and a meta-analysis reported that thyroid replacement therapy has a beneficial effect on lipid profiles, underscoring the potential for intervention to rectify lipid abnormalities associated with hypothyroidism (43). Moreover, approximately half of the mothers with hypothyroidism in our study required levothyroxine dosage adjustments after laboratory assessments, which may contribute to correcting LDL-C abnormalities over time. Thyroid hormones facilitate hepatic LDL-C uptake through upregulation of LDL receptors, thereby enhancing LDL-C clearance; reduced thyroid hormone levels may decrease LDL receptor expression, resulting in the accumulation of LDL-C in the bloodstream. Furthermore, altered lipoprotein dynamics contribute to dysregulated lipid profiles (7, 37).

Motil et al. first reported an association between thyroid hormones (T3 and T4) and milk production (44). Extending this line of inquiry, we evaluated correlations between HM FAs and thyroid markers and found that FT4 was associated with multiple altered milk FAs, while TSH and FT3 showed more limited associations. Zhao et al. also reported associations between thyroid function and serum SFAs, including an inverse relationship between FT4 and C23:0, consistent with our findings (45).

Although infant clinical outcomes were not assessed, the observed HM lipid profile, including high n-6/n-3 ratios across the cohort and higher levels of TFAs such as elaidic acid in the hypothyroid group, may be relevant for infant nutrition. Prior literature has linked higher n-6 PUFA exposure and elevated n-6/n-3 ratios with less favorable neurodevelopmental outcomes, increased adiposity, and allergic manifestations. Theoretically, this pro-inflammatory profile, combined with the presence of industrial TFAs like elaidic acid, which interferes with essential fatty acid metabolism, could predispose infants to metabolic dysregulation (40, 46, 47). Nevertheless, extrapolation to infant outcomes should be cautious because HM is a complex biological fluid and because observational associations may reflect shared genetic and environmental factors. In addition, family dietary patterns may contribute to infant exposures beyond lactation, complicating causal inference.

This study has several notable strengths. We included 19 mothers with pre-gestational hypothyroidism, a sample size that surpasses those found in several prior studies that often included fewer than 10 participants. This larger cohort allows for more meaningful comparisons between the groups. Maternal diet, lipid profile, and anthropometric measurements were collected to reduce confounding and ensure comparability. Furthermore, HM was collected through the full expression of one breast, capturing both foremilk and hindmilk, which is crucial for a comprehensive analysis of milk composition. Thyroid function was measured using laboratory testing, enabling exploratory correlations between thyroid markers and HM FA composition. To the best of our knowledge, this is the first study to assess HM macronutrients and FA composition in relation to maternal hypothyroidism.

Several limitations should be considered. The study did not include longitudinal infant follow-up; therefore, clinical data on infant growth, neurodevelopment, or lipid profiles were not available, precluding conclusions regarding infant outcomes. Inclusion of overweight and obese mothers may improve generalizability but may introduce residual confounding. Most mothers were not exclusively breastfeeding, and cultural constraints limited 24-h sampling; therefore, single-time samples may not capture diurnal variation in HM composition. Although mothers with hypothyroidism were treated, thyroid function was not uniformly within the reference range at the time of sampling. The hypothyroid subgroup was small (*n* = 19), limiting power and potentially generalizability. Finally, because this analysis was exploratory, no correction for multiple comparisons was applied; accordingly, statistically significant findings, particularly across numerous individual FAs, should be interpreted cautiously.

Overall, these findings support a potential association between maternal hypothyroidism and selective differences in HM FA composition, including higher levels of TFAs such as elaidic acid and shifts in select PUFA and SFA species. The clinical significance of these differences remains uncertain; however, the low DHA levels and altered FA ratios observed across the cohort highlight a potential area of concern for neonatal nutrition. In settings with a high prevalence of hypothyroidism, thyroid status may be a modifiable factor in milk quality. Nevertheless, caution is warranted in translating these findings to immediate practice. Rather than immediate dietary prescriptions, future longitudinal research is needed to investigate whether integrating thyroid-aware lactation support and targeted nutritional interventions can effectively modulate these effects and improve breastfeeding outcomes.

## Data Availability

The datasets used in this study are not publicly available due to privacy and ethical restrictions. Requests to access the data should be directed to the corresponding author, Shahad Alodhaybi Shahadalodhaybi@gmail.com.
