# Correction: Impact of maternal hypothyroidism on human milk macronutrient content and fatty acid composition: a prospective cohort study

**DOI:** 10.3389/fnut.2026.1864785

**Published:** 2026-05-14

**Authors:** Shahad Alodhaybi, Manal Abdulaziz Binobaed, Rasha Homoud AlAnazi, Nora Elwehedy, Muneera Baraja, Fatimah Yousef Aljawoan, Waleed Tamimi, Lamia Mohammed Elamin, Azza Madkhali

**Affiliations:** 1 Department of Food Science and Nutrition, King Saud University, Riyadh, Saudi Arabia; 2 Department of Food Science and Nutrition, King Saud University, Riyadh, Saudi Arabia; 3 Pediatric Clinic, Ministry of National Guard Health Affairs, Riyadh, Saudi Arabia; 4 King Abdulaziz Medical City, King Abdullah International Medical Research Center, King Saud bin Abdulaziz University for Health Sciences, Riyadh, Saudi Arabia; 5 Family Medicine, Ministry of National Guard Health Affairs, Riyadh, Saudi Arabia; 6 Dent Medical Center, Riyadh, Saudi Arabia; 7 Breastfeeding Association, Riyadh, Saudi Arabia; 8 Laboratory Medicine, Ministry of National Guard Health Affairs, Riyadh, Saudi Arabia; 9 Consultant, Obstetrics and Gynecology, Ministry of National Guard Health Affairs, Riyadh, Saudi Arabia

**Keywords:** fatty acid composition, human milk, hypothyroidism, lactation, Saudi Arabia, trans fatty acids

Author Shahad Alodhaybi was erroneously assigned as corresponding author. The correct corresponding author is Azza Madkhali (madkhalia@mngha.med.sa).

The original version of this article has been updated.

