# Supplementary material for: Impact of maternal hypothyroidism on human milk macronutrient content and fatty acid composition: a prospective cohort study
Source: Front Nutr. 2026 Feb 10;13:1769344. doi: 10.3389/fnut.2026.1769344 (PMC12929449; doi:10.3389/fnut.2026.1769344)
Supplement: Supplementary file 1 [file Supplementary_file_1.docx]

***Supplementary material***

# **Table S1: Comparison of maternal and infant anthropometrics between mothers with and without hypothyroidism.** Data are mean (SD) or median (IQR) as appropriate. p values for continuous variables are from two-sample t test or Mann–Whitney U test, depending on distribution. BMI=body mass index. Maternal postpartum anthropometrics were measured at the time of milk sample collection.

| **Anthropometric characteristics** | **Non-hypothyroid (N=30)** | **hypothyroid (N=19)** | **P value** |
| --- | --- | --- | --- |
| **Maternal** Pre-pregnancy BMI (kg/m^2^) | 26.79 (4·51) | 28.79 (3·55) | 0.11 |
| **Maternal postpartum** **anthropometrics** | | | |
| BMI (kg/m^2^) | 29.63 (4·71) | 30.26 (3.67) | 0.63 |
| Fat mass (%) | 39.48 (5.12) | 40.56 (4.39) | 0.42 |
| Water (%) | 45.11 (3.50) | 43.46 (3.92) | 0.13 |
| Muscle mass (kg) | 42.10 (40.00– 45.78) | 41.70 (38.70– 44.90) | 0.44 |
| Maternal height (cm) | 160.00 (156.00–161.25) | 158.00 (154.00–160.00) | 0.25 |
| **Infant** **anthropometrics** | | | |
| Infant birth weight (kg) | 3.09 (0.42) | 2.94 (0.42) | 0.23 |
| Infant 2-month weight (kg) | 5.06 (0.64) | 5.09 (0.65) | 0.84 |
| Infant 2-month height (cm) | 56.30 (54.93–58.50) | 55.00 (53.30– 57.00) | 0.22 |
| Infant 2-month head circumference (cm) | 38.87 (1.51) | 38.95 (1.31) | 0.84 |

**Table S2: Comparison of maternal dietary intake from 24 h recall between mothers with and without hypothyroidism.** Data are mean (SD) or median (IQR). p values for continuous variables are from two-sample t test or Mann–Whitney U test, depending on distribution. SFA=saturated fatty acids. MUFA=monounsaturated fatty acids. PUFA=polyunsaturated fatty acids.

| **Dietary intake** | **Non-hypothyroid (n=30)** | **Hypothyroid (n=19)** | **P value** |
| --- | --- | --- | --- |
| Energy (kcal/day) | 1674.77(329.65) | 1792.00 (312.86) | 0·22 |
| Carbohydrates (g/day) | 202.00 (53.80) | 204.53 (53.35) | 0.87 |
| Protein (g/day) | 63.00 (51.75–76.50) | 69.00 (54.00–80.00) | 0.38 |
| Fat (g/day) | 68.50 (53.75–82.25) | 74.00 (68.00–83.00) | 0.13 |
| SFA (g/day) | 18.94 (14.86– 25.19) | 19.82 (15.30–26.80) | 0.97 |
| MUFA (g/day) | 19.80 (6.77) | 20.66 (5.51) | 0.65 |
| PUFA (g/day) | 13.51 (5.80) | 16.78 (5.16) | 0.05 |
| Omega-3 (g/day) | 1.04 (0.45) | 1.18 (0.50) | 0.31 |
| Omega-6 (g/day) | 11.67 (5.28) | 14.50 (5.09) | 0.07 |
| Omega-6/ omega-3 | 10.00 (8.75–14.00) | 13.00 (9.00–16.00) | 0.27 |
| Trans fat (g/day) | 0.16 (0.03–0.46) | 0.12 (0.01–0.38) | 0.98 |
| Cholesterol (mg/ day) | 279.06 (118.09) | 295.59 (137.09) | 0.66 |
| Fat (kcal/day) | 641.47 (158.55) | 687.81 (135.81) | 0.30 |
| SAT (kcal/day) | 170.48 (133.16–226.71) | 178.62 (143.50–242.42) | 0.57 |

# **Table S3: Patterns of levothyroxine therapy and thyroid-stimulating hormone (TSH) status among mothers with hypothyroidism.** Data are n (%). All hypothyroid mothers were treated with levothyroxine. TSH=thyroid-stimulating hormone.

| **Hypothyroid mothers’ status** | n (%) |
| --- | --- |
| **Medication use** | |
| Levothyroxine intake during the last 4 weeks  Yes  No | 18 (94.7%)  1 (5.3%) |
| frequency of levothyroxine intake in the last 4 weeks  Never  Less than 3 times a week  Daily | 1 (5.3%)  3 (15.8%)  15 (78.9%) |
| Levothyroxine taken before laboratory tests  Yes  No | 11 (57.9%)  8 (42.1%) |
| Started medication during pregnancy  Yes  No | 5 (26.3%)  14 (73.7%) |
| Dose increase required during pregnancy  Yes  No | 14 (73.7%)  5 (26.3%) |
| **Laboratory assessment** |  |
| TSH at time of milk collection  Below reference range  Within reference range  Above reference range | 7 (36.8%)  9 (47.4%)  3 (15.8%) |

# **Table S4: TPOAb statues of hypothyroid and non-hypothyroid mothers.** Data are n (%). Values are compared between groups using χ² test. thyroid peroxidase antibodies (TPOAb)

| **TPOAb** | **Non-hypothyroid (N=30)** | **hypothyroid (N=19)** | **P value** |
| --- | --- | --- | --- |
| Negative | 19 (63.3%) | 1 (5.3%) | 0.001 |
| Positive | 11 (36.7%) | 18 (94.7%) |  |

# **Table S5: Results from ANCOVA analyses controlling for breastfeeding exclusivity.** The model evaluates the effect of maternal thyroid status (predictor) on fatty acid concentrations while controlling for breastfeeding exclusivity (exclusive vs. mixed) as a covariate. F represents the F-statistic; Adjusted R² represents the proportion of variance in fatty acid levels explained by the model. Bold p-values indicate statistical significance ($p < 0.05$). Degrees of freedom (df) for all comparisons were (1, 46).

| **Dependent Variable (Fatty Acid)** | **Thyroid Status (Predictor)** | | **Breastfeeding Exclusivity (Covariate)** | | **Model Fit** |
| --- | --- | --- | --- | --- | --- |
|  | **F** | **p-value** | **F** | **p-value** | **Adjusted R²** |
| C18:1 n-9t (Elaidic acid) | 15.118 | **<0.001** | 0.001 | 0.970 | 0.216 |
| C19:1 (7-Nonadecenoate) | 15.000 | **<0.001** | 2.690 | 0.108 | 0.235 |
| C20:3 n-3 (Eicosatrienoic acid) | 10.240 | **0.002** | 0.230 | 0.632 | 0.147 |
| C11:0 (Undecanoate) | 11.390 | **0.002** | 0.400 | 0.529 | 0.165 |
| C23:0 (Tricosanoate) | 10.040 | **0.003** | 0.490 | 0.488 | 0.146 |
| C22:2 n-6 (Docosadienoic acid) | 6.570 | **0.014** | 0.720 | 0.400 | 0.107 |
| C16:1 (Palmitoleate) | 7.005 | **0.011** | 0.306 | 0.583 | 0.105 |
| C21:0 (Heneicosanoate) | 6.040 | **0.018** | 0.280 | 0.601 | 0.087 |
| C22:5 n-3 (Docosapentaenoic acid) | 5.510 | **0.023** | 0.110 | 0.744 | 0.068 |
| C19:1 (10-Nonadecenoate) | 4.190 | **0.046** | 1.840 | 0.182 | 0.070 |

# **Table S6: Pearson correlation coefficient (r) between thyroid hormones (TSH, FT4, FT3) and human milk fat and fatty acids (n=49).** Values are Pearson correlation coefficient (r), two tailed tests. Significance: P<0.05 (*) and P<0.01 (**). Fatty acids not included in the figure.

| Fatty acids in human milk | TSH | FT4 | FT3 |
| --- | --- | --- | --- |
| C6:0 (Hexanoate) | 0.063 | 0.202 | 0.005 |
| C8:0 (Octanoate) | -0.095 | 0.053 | 0.141 |
| C10:0 (Decanoate "caprate") | -0.026 | -0.147 | 0.112 |
| C12:0 (Laurate) | 0.026 | -0.301* | 0.058 |
| C13:0 (Tridecanoate) | 0.146 | -0.098 | -0.109 |
| C14:0 (Myristate) | 0.060 | -0.293* | -0.014 |
| C15:0 (Pentadecanoate) | 0.126 | 0.155 | -0.043 |
| C16:0 (Palmitate) | 0.073 | -0.179 | -0.202 |
| C17:0 (Heptadecanoate) | 0.077 | -0.021 | 0.090 |
| C18:0 (Stearate) | 0.243 | 0.028 | 0.067 |
| C20:0 (Arachidate) | 0.092 | -0.031 | 0.139 |
| C22:0 (Behenate) | 0.036 | -0.283* | 0.159 |
| C24:0 (Lignocerate) | -0.001 | -0.051 | 0.057 |
| C14:1 (Myristoleic acid) | 0.059 | 0.138 | -0.084 |
| C16:1 (Palmitoleate) | -0.137 | 0.033 | -0.109 |
| C17:1 (Heptadecenoic acid) | -0.002 | -0.213 | -0.007 |
| C18:1 n-9 (Oleic acid) | -0.104 | 0.129 | 0.039 |
| C18:1 n-7 (Vaccenic acid) | -0.149 | 0.243 | -0.054 |
| C20:1 n-9 (Gondoic) | -0.015 | 0.104 | 0.187 |
| C22:1 n-9 (Erucate) | -0.044 | 0.085 | 0.185 |
| C24:1 n-9 (Nervonate) | -0.098 | 0.025 | 0.021 |
| C18:3 n-6 (γ-Linolenic acid, GLA) | -0.235 | 0.459** | 0.047 |
| C18:4 n-3 (Stearidonic acid, SDA) | -0.112 | -0.186 | -0.029 |
| C20:2 (Eicosadienoic acid) | -0.003 | 0.140 | 0.127 |
| C20:3 n-6 (Dihomo-γ-linolenic acid, DGLA) | -0.118 | 0.166 | 0.082 |
| C20:4 n-6 (Arachidonic acid, ARA) | -0.190 | 0.246 | 0.054 |
| C22:2 n-6 (Docosadienoic acid) | -0.016 | 0.344* | -0.066 |
| C14:1 (Myristoleic acid, trans) | 0.535** | -0.318* | 0.069 |
| C15:1 (Pentadecenoic acid, trans) | 0.446** | -0.259 | 0.129 |
| C17:1 (Heptadecenoic acid, trans) | 0.051 | 0.238 | -0.076 |
| C18:1 (Vaccenic acid, trans) | -0.102 | -0.277 | -0.149 |
| C18:2 n-6 (Linolelaidic acid, trans) | -0.045 | -0.074 | -0.038 |
| C20:1 (Gondoic acid, trans) | 0.037 | -0.039 | -0.141 |
